# Repeated successful surgical rescues of early and delayed multiple ruptures of ventricular septum, right ventricle and aneurysmal left ventricle following massive biventricular infarction

**DOI:** 10.1186/1749-8090-1-30

**Published:** 2006-09-28

**Authors:** Pankaj Kaul

**Affiliations:** 1 Consultant Cardiac Surgeon, Yorkshire Heart Centre, Leeds General Infirmary, Great George Street, Leeds, LS1 3EX, UK

## Abstract

A 58 year old man underwent 6 surgical interventions for various complications of massive biventricular myocardial infarction over a period of 2 years following acute occlusion of a possibly "hyperdominant" left anterior descending coronary artery. These included concomitant repair of apicoanterior post-infarction VSD and right ventricular free wall rupture, repeat repair of recurrent VSD following inferoposterior extension of VSD in the infarcted septum 5 weeks later, repair of delayed right ventricular free wall rupture 4 weeks subsequently, repair of a bleeding left ventricular aneurysm eroding through left chest wall 16 months thereafter, repair of right upper lobe lung tear causing massive anterior mediastinal haemorrhage, mimicking yet another cardiac rupture, 2 months later, followed, at the same admission, 2 weeks later, by sternal reconstruction for dehisced and infected sternum using pedicled myocutaneous latissimus dorsi flap. 5 years after the latissimus myoplasty, the patient remains in NYHA class 1 and is leading a normal life.

## Background

Surgery is required immediately following acute myocardial infarction for the mechanical complications like VSD, mitral regurgitation and free wall rupture of ventricle, mostly left, and subsequently for left ventricular aneurysm repair with or without coronary artery bypass grafting, or coronary bypass grafting alone. An acute occlusion of an anterior descending coronary artery presents with an anterior myocardial infarction and occasionally with an anterior post-infarction VSD and rarely with a free wall rupture of left ventricle. The mortality for emergency or salvage surgery for either post-infarction VSD or free wall rupture continues to be high. Concomitant repair of post-infarction VSD and free wall rupture has a prohibitively high mortality. Described below is the successful outcome in a patient who sustained a massive biventricular infarction following an acute occlusion of a possibly "hyperdominant" left anterior descending coronary artery. This led to a large post-infarction anterior VSD and, surprisingly, a right ventricular free wall rupture requiring a concomitant repair of the two pathologies. This was followed by a number of delayed catastrophic complications of biventricular infarction, over a period of 2 years, involving ventricular septum and the two ventricles involved requiring multiple salvage procedures. Each of these successful surgical rescues made it possible for the next event in the natural history of massive biventricular infarction with its early and delayed complications to unfold.

## Case report

**Figure 1:**
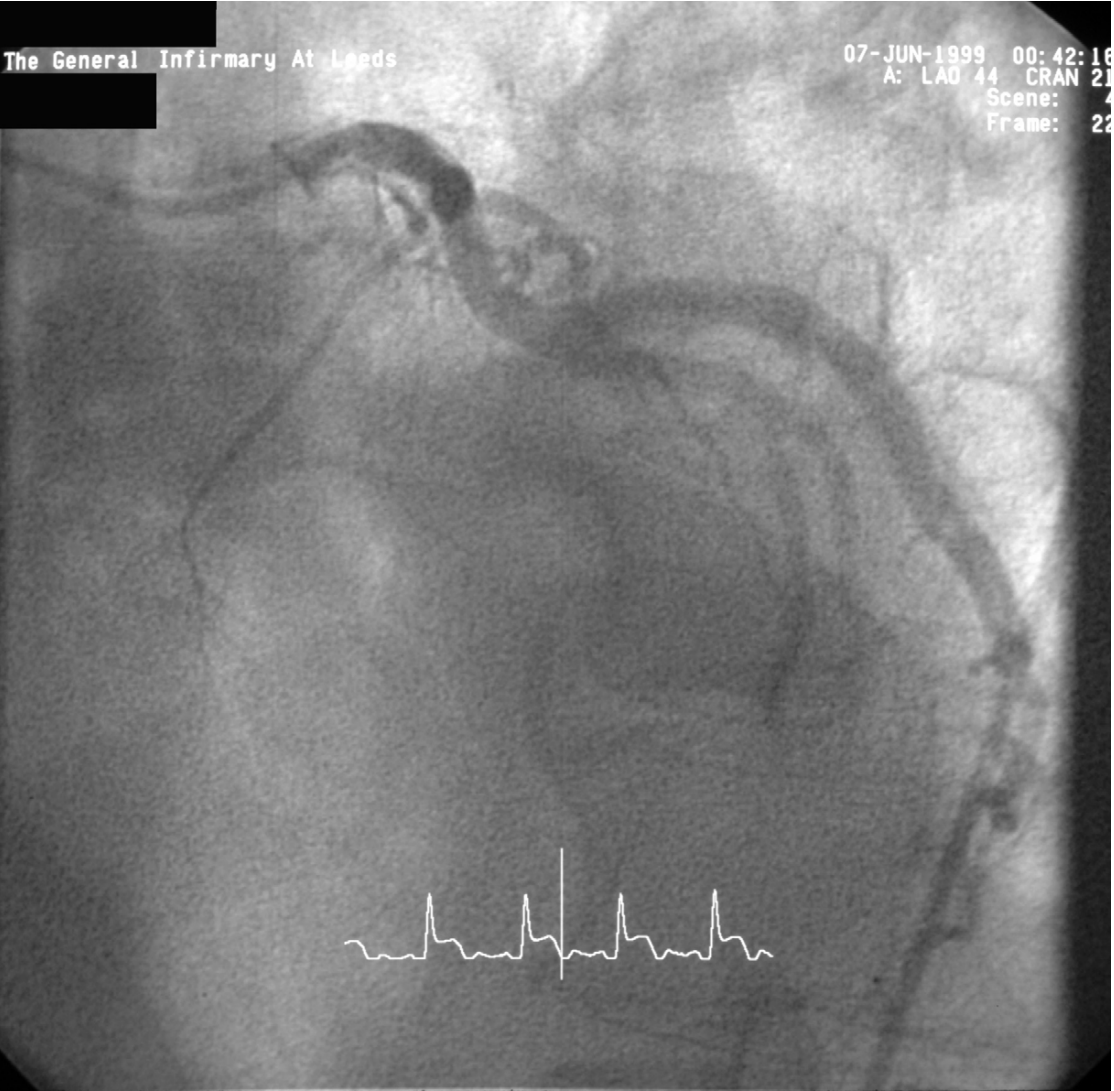
Left coronary angiogram (LAO view) showing occluded LAD.

**Figure 2:**
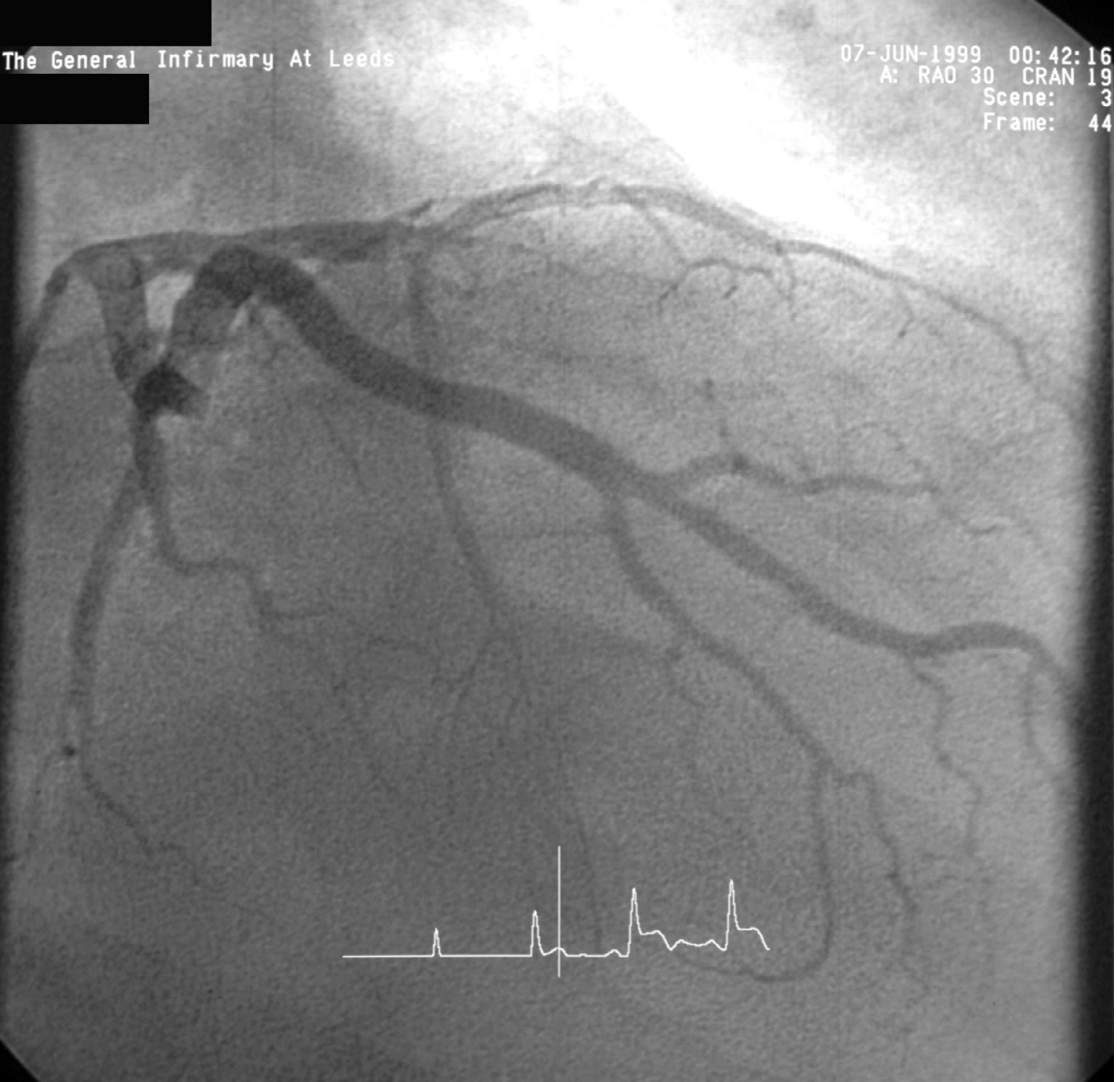
Left coronary angiogram (RAO view) showing an occluded LAD just beyond the first septal and the first diagonal branches.

A 58 year old man presented to Accident and Emergency with acute chest pain on 6/6/99. Examination revealed tachycardia, a loud pansystolic murmur along left sternal edge and bilateral basal crackles. Chest x-ray suggested pulmonary oedema. ECG demonstrated ST elevation in leads 1, AVL, V1–V5. He was thrombolysed with streptokinase. Transthoracic echo revealed an apicoanterior VSD with a significant shunt and akinesia of the anterior left ventricular wall. Coronary angiography demonstrated normal left main stem, occlusion of LAD just beyond the first septal and first diagonal (figs 1 and 2), and normally distributed and disease free circumflex and right coronary arteries. RCA was a normal sized vessel which supplied PDA which fell short of the apex. Circumflex artery, too, was a normal sized vessel with an average sized OM branch. The first diagonal was a good sized vessel which reached almost ¾ of the way down to apex. He was diagnosed to have anteroseptal myocardial infarction secondary to an occluded LAD, and a large anteroapical VSD. An IABP was introduced percutaneously. He continued to deteriorate clinically and became anuric and went into shock. On emergency median sternotomy on 7/6/99, the operative findings included hemopericardium, large anteroapical infarct of LV and anterior surface of RV, free wall rupture of infarcted RV wall just below RVOT and large 3 cm VSD in the anterior septum. Under CP bypass, moderate hypothermia and antegrade cardioplegic arrest, left ventriculotomy was made lateral to the diagonal branch of LAD. The septal myocardium surrounding the VSD was necrotic and mushy. The VSD was closed using a Teflon patch using interrupted pledgetted sutures placed well beyond the unhealthy septal myocardium surrounding the VSD in the inferior half and incorporating the superior half in sutures used for LV closure, which was done in two layers using Teflon bolsters on either side of ventriculotomy. The ruptured infarcted area of RV was incorporated in the suture line with the suture for ventriculotomy closure coming out well beyond the macroscopically identifiable right limit of RV infarction. All sutures in septum, left ventricle and right ventricle were placed through grossly uninfarcted myocardium. The distal half of the diagonal branch had to be incorporated in the ventriculotomy repair as it was involved in the infarcted myocardium. Bypass was discontinued easily in sinus rhythm on a small dose of ionotropes and IABP. Postoperative echo on 8/6/99 showed no residual VSD. He made satisfactory progress and was transferred to the ward on 6th postop day and went home on 17/6/99, 10 days following his surgery.

He represented on 14/7/99, 5 weeks after his previous VSD repair, from which he had been making satisfactory progress at home, with a 3 day history of progressively worsening shortness of breath. Clinical picture was that of pulmonary edema. TTE showed large VSD with 3:1 L-R shunt and moderate left ventricular function. He was taken to theatre and IABP introduced percutaneously through right groin. Femorofemoral bypass was instituted through left groin and patient cooled to 17°C. Secondary median sternotomy was made and heart dissected out with considerable difficulty. Aorta was not cross clamped and the previous ventriculotomy was reopened with the heart in VF. There was a recurrent VSD occupying a position inferoposterior to the Teflon patch with a serpiginous extension of septal infarct inferiorly and posteriorly over a wide area well beyond what was seen at the first operation, extending almost to the inlet of the trabecular septum. This was repaired using a much larger Teflon patch, anchoring the superior margin of the patch to the inferior margin of the previous patch and stitching the inferior margin well beyond the infarcted septum. This effectively excluded the entire area of the infarcted septum well beyond the VSD. The anterior wall of right ventricle in its left half was scarred and thinned out. Ventriculotomy was closed in two layers patient rewarmed, and bypass discontinued after heart defibrillated spontaneously into sinus rhythm. Postoperative echo on 21/07/99 showed a small L-R shunt and significantly impaired biventricular function. Despite this patient made a surprisingly quick recovery and was transferred to the ward on day 4 and home on 30/07/99.

On 06/08/99 he represented to the Accident and Emergency with a 4 cm subcutaneous swelling in the lower part of the sternal wound. This was incised, a small amount of pus drained which was sent for bacterial culture and the wound managed with daily dressings. On 11/08/99 when he was making preparations for his discharge, he suddenly collapsed after a bout of exsanguinating haemorrhage from the lower part of his sternal wound. He was resuscitated and transferred to theatre while bleeding was partially controlled by hand pressure. Femorofemoral bypass was instituted and the sternum reopened with an oscillating saw and heart dissected out. There was a 3 cm by 3 cm rupture of the right ventricular free wall about 5 cm inferior to the pulmonary valve and 5 cm to the right of the previous ventriculotomy repair. This was repaired directly using Teflon bolstered interrupted mattress sutures, on bypass, without cross clamp and with the heart beating. Bypass was discontinued easily and patient made satisfactory postop recovery. A TTE revealed dyskinetic anterior wall with moderate function elsewhere and no residual VSD and he was discharged home on 20/08/99.

**Figure 3:**
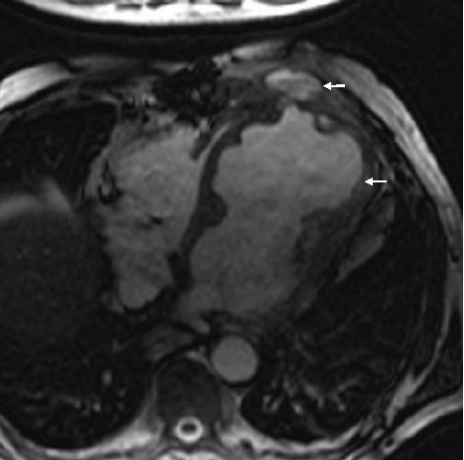
MR scan showing left ventricular aneurysm (arrow) and blood from contained aneurysmal rupture lying under pectoralis minor (small arrow).

**Figure 4:**
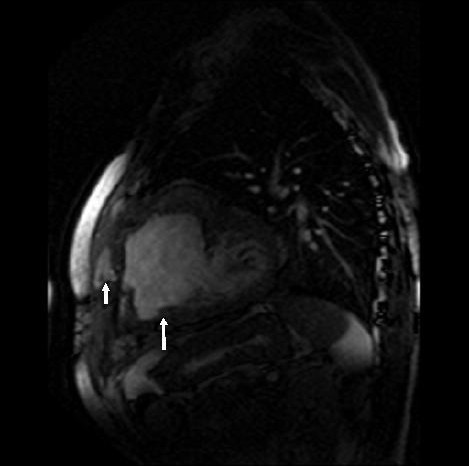
MR scan, lateral view, showing LV aneurysm (large arrow), contained aneurysmal rupture(small arrow) and small LV cavity (posterior to LV aneurysm).

**Figure 5:**
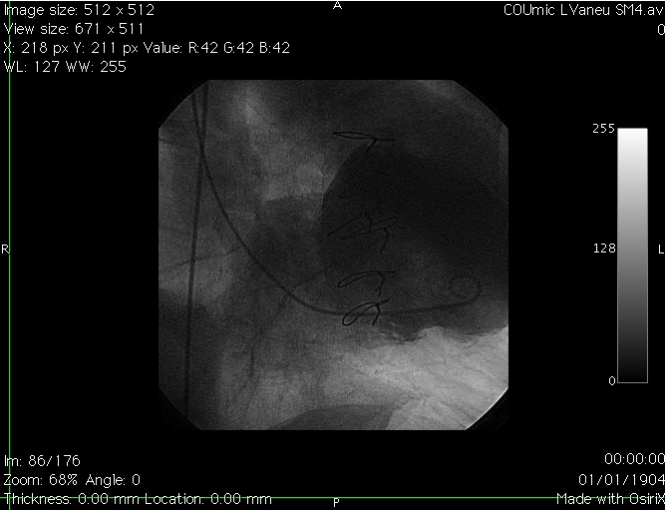
Left ventricular angiogram showing large aneurysm (catheter tip inside the aneurysm).

Except for a grumbling sternal wound for which he underwent removal of top three sternal wires, patient remained symptomatically well and led a healthy outdoor life for the next more than a year. On 01/11/00, 14 months after his last admission, however, a CT scan showed a large left ventricular aneurysm, involving the entire anterior and apical surface of left ventricle and including the previous ventriculotomy repair. This had partially eroded through left anterior chest wall and was lying under left pectoralis minor. An MR scan on 02/11/00 revealed a large left ventricular aneurysm extending from diaphragm to the level of pulmonary bifurcation with extension of aneurysm through intercostal space into left pectoralis minor. There was an area of fluid collection under the pectoralis minor which probably was altered blood secondary to a contained rupture of the aneurysm. (Figs 3 and 4). A left ventriculogram on 23/11/00 confirmed a large left ventricular aneurysm involving more than half of the total left ventricular cavity with moderate residual function, but with no evidence of active leak from the aneurysm (Fig 5). It was not clear if LV aneurysmectomy would leave sufficient LV mass to sustain life in view of the sheer proportion of LV involved in aneurysmal change. Heart transplantation was clearly not an option in light of sternal infection and multiple previous surgeries.

**Figure 6:**
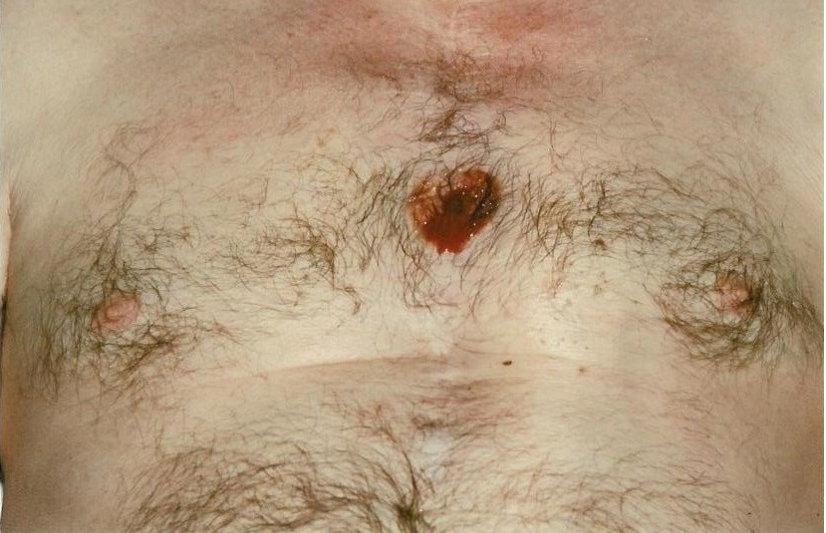
Left ventricular aneurysm with impending cutaneous rupture.

**Figure 7:**
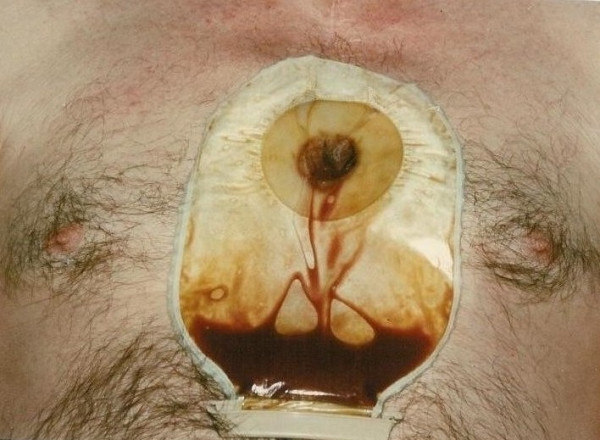
Ruptured left ventricular aneurysm – a left ventriculocutaneous fistula.

**Figure 8:**
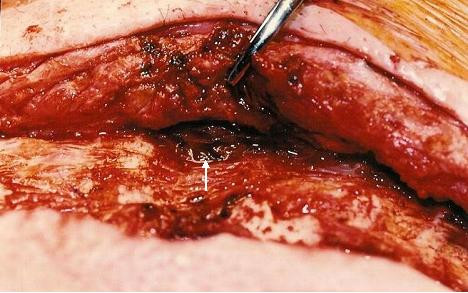
Pectoralis major reflected to reveal left ventricular aneurysm eroding through intercostal space (arrow).

On 26/12/00, however, he presented with a tense pulsatile mass in the third left intercostal space extending to a sinus under mid sternum (Fig 6) followed later by slow bloody discharge from the sternum (Fig 7). Faced with the prospect of exsanguinating haemorrhage from the chest wall eroding aneurysm, it was elected to surgically intervene. The sternal skin was incised, the left pectoralis major was reflected upto mid axillary line and the left ventricular aneurysm eroding through the third intercostal space was exposed (Fig 8). Femorofemoral bypass was instituted and secondary median sternotomy performed. Heart was dissected out with difficulty and patient cooled to 18°C. The aneurysm involved almost the entire anterior and apical surfaces of left ventricle and included the previous ventriculotomy repair. The aneurysm was completely excised, the Teflon of the previous ventriculotomy repair and the clots within the aneurysm removed and the new left ventricle reconstituted employing linear closure using long strips of Teflon on either side of ventriculotomy. Aortic cross clamp or circulatory arrest was not necessary. Patient made good recovery and was weaned off ventilation, ionotropes and IABP over the next 3 days. Excised ventricular aneurysmal tissue grew coagulase negative staph and staph epidermidis for which he received for the next 4 weeks followed by oral flucloxacillin for next 2 weeks. On 02/01/01, a repeat MR scan showed significantly improved LV, no residual aneurysm and no residual VSD. He was discharged home on 02/01/01.

**Figure 9:**
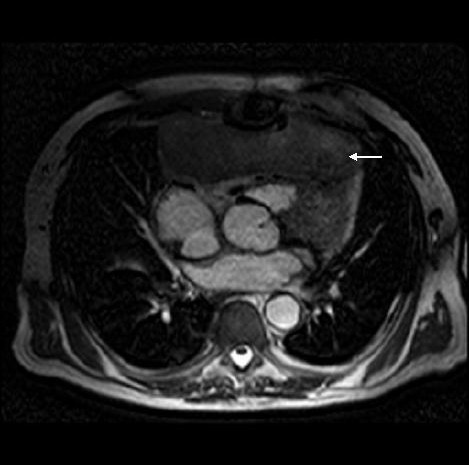
MR scan showing large anterior mediastinal collection of clot and blood (arrow).

He remained well till 25/02/01 when he presented with 2 day history of shortness of breath, cough and haemoptysis. Examination revealed an inflamed and tender swelling in the lower sternal area. MR scan on 26/02/01 demonstrated a large anterior mediastinal collection (Fig 9). It was not clear from the films if there was any communication between either ventricle and anterior mediastinal clot or indeed the tracheobronchial tree. Meanwhile on 01/03/01, the subcutaneous swelling progressively increased in size and was close to bursting. It was felt this was once again an impending rupture of heart and therefore, the regional transplantation team was contacted who felt heart transplantation would be inappropriate. The patient was keen to have something done and he was transferred to theatre on 01/03/01.

The subcutaneous swelling over the lower sternum was aspirated and this was frank blood. Femorofemoral bypass was established through left groin and patient cooled to 28°C. Left anterolateral thoracotomy was made through 4th space to avoid going through the sternum the 5^th ^time. The whole of the anterior mediastinum and the upper third of right hemithorax were filled with clot. After the fresh clot was evacuated from the mediastinum, we were pleasantly surprised to find that heart was pristine clear with healthy previous suture line and nothing to suggest rupture. Instead after sucking out clots from right hemithorax, it was clear there was ongoing fresh bleeding from the apex of right lung. Visualisation of this was not possible from left thoracotomy and therefore sternum was reopened the 5^th ^time. There was a large vascular adhesion at the apex of the right lung that had torn and both the pleural and parenchymal ends were bleeding actively. There was an associated tear in the upper lobe of lung with a substantial air leak. The vascular adhesions were secured, bleeding controlled and lung tear controlled with difficulty with sutures and glue. Bypass was discontinued easily but the sternum looked quite unhealthy with substantial loss of bone and multiple fractures and I closed it with heavy ethibond in preparation for what I felt would be an inevitable plastic repair in future.

**Figure 10:**
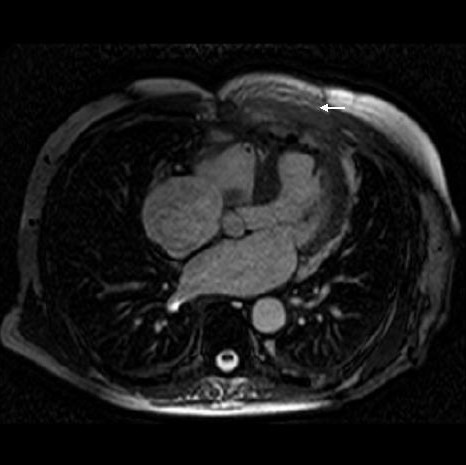
Postoperative MR scan showing latissimus dorsi myocutaneous flap (arrow) covering the heart after sternectomy. There is no residual VSD and left and right ventricles are of normal size.

Patient made a good haemodynamic recovery but expectedly the sternum did not heal and there was a persistent air leak from the right pleural drain. On 16/03/01, he was returned to theatre. Radical debridement of most of necrotic sternum and 3^rd ^to 7^th ^costal cartilages was done. The patient was turned on the side and a large 10 cm × 27 cm musculocutaneous latissimus dorsi flap was raised including a patch of lumbar fascia. Flap was passed under skin into sternal region. The flap was inserted into the resultant sternal space, lumber fascia used to close off the abdominal cavity and a muscle tongue directed into the apex of right pleural cavity to seal off the still persistent air leak. Patient was re-explored for bleeding underneath the patch on the same evening and haemostasis secured from an arterial spurter from the bleeding bone. Postop course was complicated by persistent air leak which eventually stopped on 18/04/01. Thereafter he made good recovery and was discharged home on 19/04/01 on long term antibiotics. His postoperative MR scan showed no residual VSD, no LV or RV aneurysm and a healthy latissimus myoplasty (Fig 10)

**Figure 11:**
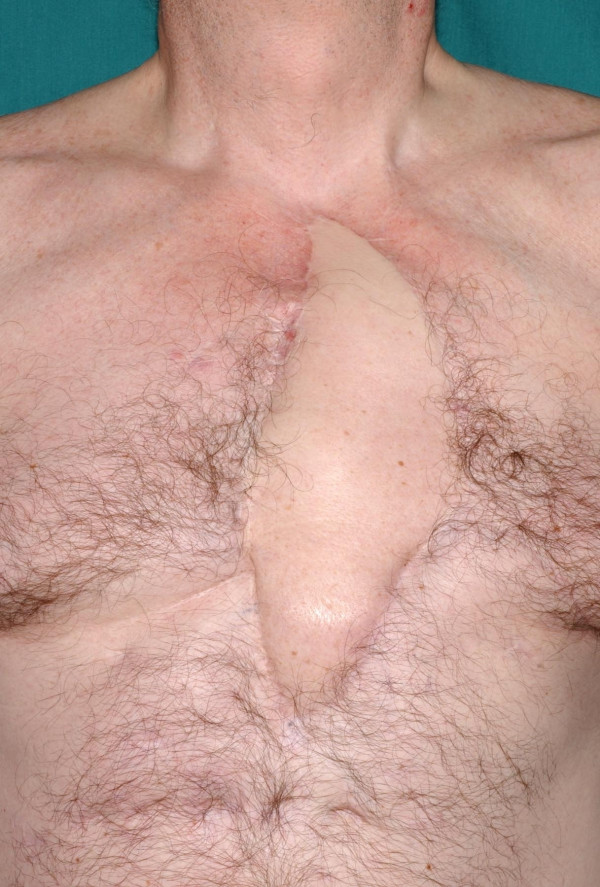
Sternal scar 5 years following sternectomy and reconstruction with latissimus dorsi myocutaneous flap.

Patient remains well and has required no hospitalisations or operations since. His antibiotics were stopped on 04/03/02. He is seen in outpatient clinic at 6 monthly intervals and leads a purposeful life and remains asymptomatic with a well healed sternal scar (Fig 11).

## Discussion

Post infarction VSD complicates approximately 1% to 2% of patients after myocardial infarction and accounts for 5% of early deaths after myocardial infarction [1,2]. The quantum of right ventricular infarction is much greater in hearts with septal defects than in those without septal defects [3]. A substantial right ventricular infarction is common with postinfarction VSDs and contributes significantly towards cardiogenic shock and subsequent death [3]. However, right ventricular infarction is associated much less often with anterior VSDs than with inferior VSDs [3]. Anterior VSDs constitute 60% of all postinfarction VSDs, usually as a complication of first acute MI, in patients with an acute occlusion of LAD with a relatively poor collateral circulation with or without a concomitant right coronary stenosis [[4-6] and [7]]. Amongst anterior VSDs, there is evidence that a rupture located more proximally is associated with a worse prognosis [8]. Acute rupture of the free wall of the LV complicates 4% to 24% of patients with acute MI.[9] and is the second most common cause of death following acute infarction, next only to myocardial pump failure, responsible for as many as 20% of early deaths [10]. It is usually categorised as acute blowout generally not amenable to treatment, sub acute rupture presenting commonly with cardiac tamponade and a chronic rupture with false aneurysm [11]. In an autopsy series of patients of deaths from AMI, cardiac rupture was present in 30.7% patients, with LV rupture in 98% (anterior wall 45%, posterior wall 38%, lateral wall 9% and apex 6%) and RV rupture in 2% [12]. Rupture of both ventricular free wall and septum is an unusual event. In a large series of 3284 patients with AMI, double rupture was observed in 0.30% [10] patients only, but in 3% of those with free wall rupture, and, interestingly, in 16.1% of those with ventricular septal rupture [13]. A ventricular septal rupture in the apical region was the greatest risk factor for free wall rupture, although age, hypertension, ionotropes, thrombolytic agents, delayed reperfusion and reinfarction play a subsidiary role [13]. However, an RV rupture associated with an anterior postinfarction VSD is an extremely rare event with a few isolated case reports [14,15] although combined septal and biventricular free wall rupture repair has been successfully reported once [16].

Our patient presented with an anteroseptal and right ventricular infarction and pulmonary oedema with a large left to right shunt secondary to a large anteroapical VSD. There was a sub acute oozing type of RV rupture with hemopericardium, the site of rupture being in the infarcted RV anterior wall below the outflow tract. The rupture probably occurred just before his transfer to theatre, as his echo on admission showed only VSD and no pericardial effusion or tamponade, and would correspond to the acutely deteriorated clinical state of anuria and shock just before surgery. Association of anterior VSD with right ventricular rupture is an extremely rare event, having been previously reported only twice. This is not surprising in light of the fact that, in most individuals, LAD supplies only small branches to the right ventricular free wall, one of the larger ones of which anastomoses with the infundibular branches of the proximal right coronary artery forming the loop of Viuessens, which can be an important route of collateral blood supply. The fact that acute occlusion of LAD in our patient, even though it was beyond the first septal, led to a massive biventricular infarction, VSD, RV free wall rupture and delayed LV aneurysm and rupture points towards the "hyperdominant"role it played in the blood supply of this heart. Arguably, it might have supplied important branches to right ventricle in the absence of a good blood supply from RCA. The fact that PDA from RCA fell well short of apex leads one to believe that LDA, before occlusion, went all the way round the apex and supplied distal posterior ventricular septum as well.

The principles underlying surgery for postinfarction VSD include: 1) avoiding multiorgan damage by expeditious institution of cardiopulmonary bypass 2) transinfarct left ventriculotomy 3) a variable degree of left, right and septal infarctectomy 4) generous use of prosthetic material in closure of VSD and ventriculotomy [17].

Over the last 6 years, there has been a move away from infartectomy. The rationale for infartectomy of the ventricles was a) the desirability of avoiding delayed aneurysm formation or rupture at the infarcted ventricular suture line b) avoiding tearing of sutures through the ventricular suture line c) improving exposure of the VSD [17]. The purported rationale of infartectomy of the septum was the facilitation of placement of full thickness sutures through indisputably viable septum. There is evidence, however, that extensive infarctectomy compromises ventricular function [18]. The endeavour to place full thickness sutures through normal septal muscle, besides, is rarely successful despite septal debridement owing to complex, serpiginous and, quite often, wide involvement of the septum well beyond the visual boundaries of the VSD [19]. Securing a patch of pericardium or prosthetic material instead to the endocardium and partial thickness myocardium by means of interrupted [20,21] or running [22,23,18] sutures well beyond the physical limits of either the VSD or the surrounding septal infarct excludes the infarct, remodels the acutely infarcted left ventricle, avoids further damage to right ventricle, septum and papillary muscles through avoidance of full thickness tissue strangulating sutures and enhances survival [23].

Concomitant coronary artery surgery in patients with postinfarction VSD and single vessel disease has been shown to confer no survival benefit [24]. The results of concomitant CABG in patients with multivessel disease vary with no statistical benefit in early mortality or actuarial survival in some series [24] to significant benefit in terms of midterm mortality rates [25]. Our own results, which shall form the basis of a separate article, suggest indisputable benefit of revascularisation in patients with multivessel disease. Interestingly, postinfarction VSD has been reported in the absence of coronary artery disease [26]. Our patient underwent exclusion of infarct and VSD using a large Teflon patch which was stitched to endocardium and partial thickness myocardium using interrupted sutures. The superior 1/3 of the patch was incorporated in the Teflon bolstered ventriculotomy closure.

Other minor modifications of infarct exclusion have been described. These include use of GRF biological glue as a sealant between the patch and the infracted myocardium [27], use of acrylic glue to reinforce the pericardial patch for VSD closure [28], use of two patches, one to close VSD and the other to exclude the infarcted myocardium and GRF glue applied between the two patches [29]. There have been occasional reports of repair of postinfarction VSDs on beating hearts, the authors believing them to be of benefit in elderly patients [30,31]. postinfarction VSDs have been repaired through right atrium [32-36] and right ventricle [37,38], almost exclusively these having been repairs of inferior VSDs, although trans-atrial mid septal rupture repair has been described [32]. Failure to wean from bypass following repair of postinfarction VSD has resulted in use of femorofemoral circulatory assist with centrifugal pump which was, subsequently, successfully weaned [39,40]. Addition of a bidirectional cavopulmonary shunt to the septal repair has been described in a patient with severe right heart failure as an alternative to the use of a ventricular assist device [41]. There have been at least two reports of ventricular device failure in patients with postinfarction VSD without septal repair [42]. Right to left shunting across the VSD with hypoxic brain injury in a patient on HeartMate LVAD has been reported [43]. In a report using Hemopump axial flow device, 2 of 2 patients supported experienced lethal pump failure [44]. A greater number of patients have had left ventricular assist devices as bridges to subsequent heart transplantation [43,45,46] following either intractable heart failure or failure to wean from bypass after postinfarction VSD repair.

The repair of postinfarction VSD continues to be associated with high mortality ranging from 13.4% [47] to as high as 40.8% [48]. The NACSD 2003 reported a UK national mortality rate of 35.4% to 36.7%. Preoperative cardiogenic shock, early post infarction septal rupture, deterioration of hemodynamic status between admission and surgery [49], right ventricular dysfunction [50], left main stem involvement, renal failure, old age, posterior VSDs [51], proximal and not necessarily posterior VSD [52] and prolonged CP bypass times [53] remain important predictors of grave prognosis. Many of the predictors are surrogates for the sheer size and extent of the initial infarct. The SHOCK multi-institutional study which tracked 55 patients in cardiogenic shock with postinfarction VSD confirmed the toll this complication exacts with just 4% survival rates with medical treatment and 19% survival with surgery [54]. High operative mortality has sparked interest in percutaneous balloon [55] and percutaneous device occlusion using CardioSEAL septal occluder [56], Rashkind double umbrella [57] and the Amplatzer muscular VSD occluder device combined with multivessel stenting [58]. Results of a recent US registry report suggest immediate mortality comparable with surgery using the Amplatzer post infarct muscular VSD device (PIMVSD) in selected patients [59].

Diagnosis of postinfarction VSD is an indication of immediate operation. These patients die of multiorgan failure as a result of shock and not primarily due to cardiac failure, and supportive, expectant management deprives a vast majority of patients of the benefits of surgery [60,61]. Only 5% of patients survive medical treatment to undergo delayed in hospital surgery and this dismal statistic fails to justify the often futile attempts at prolonged "stabilisation" of these ill patients prior to surgery.

Recurrent or residual septal defects are present in 10% – 25% of patients following initial repair [63]. These are due to overlooked defects, reopening of previously intact repairs, technical difficulty in repairing large, complex VSDs, development of new septal ruptures or, rarely, due to the presence of an undiagnosed septal dissection [62]. A septal rupture only 5 weeks after previous postinfarction VSD repair can represent a new infarct but more commonly occurs as a result of the process of infarct extension. When patient is symptomatic or shunt greater than 2:1, the VSD should be closed. Trans-catheter closure offers an alternative to reoperation in many of these critically ill patients [63,64]. Right atrial approach in posterior recurrent VSDs avoids hazardous dissection often around patent coronary grafts and avoids further ventriculotomy on an already impaired ventricle [65]. Left thoracotomy and re-repair under circulatory arrest successfully has been described [66]. Small residual or recurrent VSDs benefit from reoperation through standard secondary median sternotomy but a new septal rupture with large left to right shunt is a catastrophic event with extremely high mortality. Successful orthotopic heart transplantation has been described 3 months after postinfarction VSD repair with new septal rupture [67].

Our patient presented suddenly with a new septal rupture 5 weeks after his first postinfarction VSD repair, the immediate postoperative echoes showing no shunt. He was in pulmonary oedema with a 3:1 shunt. The operation was done, through median sternotomy, under profound hypothermia without aortic crossclamp or circulatory arrest using an oversized Teflon patch to exclude a VSD extending serpiginously inferoposteriorly from the previous repair.

RV free wall rupture has diverse etiology ranging from MI [68], medistinitis [69-74], high pressure suction drainage [75], indwelling catheter injury [76], blunt cardiac trauma [77,78], closed chest resuscitation [79,80], bone fragment penetration [81], minimally invasive CABG [82], severe lipomatosis [83], severe fatty infiltration [84], chronic Chagas disease [85], Right ventricular dysplasia [86], etc. Our patient's right ventricular free wall rupture occurred 3 months after the RV infarct and was obviously predisposed to by acute regional thinning and dilatation of the previously infarcted RV wall and possibly by an infected sternotomy wound communicating with anterior mediastinum in the xiphisternal area. Patient did not exsanguinate because massive blood loss was prevented by covering the xiphisternum wound and yet he did not tamponade because substantial amount of blood did find egress through the xiphisternal wound

Over 95% of true LV aneurysms reported in English literature result from coronary artery disease and MI [87]. The rest arise from trauma [88], Chagas disease [89], sarcoidosis [90] and congenital diverticula of LV [91]. Of the aneurysms caused by coronary artery disease, 86% are anteroapical or apical, 8.6% posteroinferior and the rest 5.3% lateral. Two, three or LAD coronary artery alone were involved in 38.9%, 33.6% and 26.6% respectively [92]. False aneurysms of LV occur 5 to 10 days after occlusion most commonly of circumflex coronary artery following a contained LV rupture [87], or after contained submitral rupture of LV after MVR [93], or any surgery on LV or aortic or mitral annuli [87], and finally from septic pericarditis [94].

There are no reports of a true LV aneurysm eroding through the anterior chest wall with slow rupture in literature either with or without previous postinfarction VSD repair. Combination of large anterior infarct, 3 previous operations and possibly the absence of a radical infarctectomy at the initial surgery contributed to the event. The aneurysmectomy was done using femorofemoral bypass and systemic hypothermia to 18°C but without aortic cross clamp or circulatory arrest. A 2 cm rim of scar was left to allow a linear reconstruction but still there were concerns about the ultimate size of the residual left ventricular cavity which, however, proved adequate. An aneurysm resection or patch that leaves a poststoperative LV enddiastolic volume of about 150 mls is ideal [95]. A postoperative echo suggested reasonable function in the residual left ventricle.

The last presentation of the patient with spontaneous rupture of a vascular adhesion between upper lobe of lung and the chest wall with associated tear of the lung, filling the upper half of the right pleural cavity and the anterior mediastinum with a massive collection of blood mimicking cardiac rupture, is again without precedent in reported literature. Four previous sternotomies in emergency and salvage settings with frequent sternal wound problems would have been the substrates of a continuing and recurrent low grade mediastinitis which predisposed to this unusual site and presentation of secondary hemorrhage. In light of the absence of conclusive MR evidence to the contrary, as also the history of the frequent cardiac ruptures (thrice, first concomitant RV and VSR, second delayed RV free wall and third LV aneurysmal rupture), there perhaps was a morbid bias towards expecting the worst. What was also interesting was the fact that the bleeding took place into a space that normally would have gotten obliterated by adhesions postoperatively. Even though the right pleural cavity had been opened at the previous aneurysmal repair, it was instructive to note that even 2 months after the previous surgery, the upper half of the right pleural cavity was in free communication with anterior mediastinum.

Five sternotomies over a relatively short period of time with continuing infection set the stage for a mediastinal dead space and an infected non healing sternum and a continuing air leak. This required further operative intervention when sternectomy and mediastinal debridement was combined with a latissimus dorsi myocutaneous transposition flap, a tongue of the muscle being used to cover the lung tear. Omentum, pectoralis major, trapezius, rectus abdominis, latissimus dorsi have all been used for reconstruction after mediastinitis [96-101][101]. For reconstruction after total sternectomy with substantial infected mediastinal and right pleural space, total latissimus myocutaneous flap on its supraclavicular arterial pedicle provided the ideal bulk to obliterate the space and provide protection to the heart. Patient's oral antibiotics were continued in a rotating fashion almost a year into his last discharge from the hospital.

Patient leads a normal and productive life 5 years after his last discharge from the hospital. His latest echocardiogram shows moderate LV function, no residual VSD and no LV or RV aneurysm.

Was there a way of avoiding these multiple operations? Only an extensive infartectomy of the entire anterior ventricular septum, the entire anterior and apical surfaces of left ventricle and at least one third of the anterior wall of the right ventricle would have prevented recurrent VSD due to infarct extension, delayed rupture of thinned RV wall and sternal erosion and rupture of LV aneurysm. This would have been in the realm of experimental surgery which he would never have survived. Alternatively, a heart transplant would have been an option, but he would never have been accepted in the presence of a correctable defect.

At a time when surgeon specific mortality data for UK surgeons is freely available on the internet, and there are growing concerns that surgeons might be forced to practice more defensively to protect their results, it is cases like this that help to generate the hope that sometimes, albeit rarely, a surgeon is given a choice which, should he exercise it, shall invest his practice with some focus, some purpose that transcends cynicism and fear. At a more philosophical and ethical level, this patient taught the author how the indomitable will of one man to live can still defeat death, in its various guises. In an era when "do not resuscitate" orders are becoming more and more frequent and when manager-driven, insurance-driven, media-driven and, frequently, industry-driven considerations, ever so insidiously, under the posture of realism and practicality, compete to affect our clinical judgements and dictate our choices, it is patients like these and surgical decisions that they impel us to make, such as these, that make our surgical journeys meaningful and substantial.
